# Estimating the contribution of mortality selection to the East–West German mortality convergence

**DOI:** 10.1186/s12963-017-0151-3

**Published:** 2017-09-19

**Authors:** Tobias C. Vogt, Trifon I. Missov

**Affiliations:** 1 Max Planck Institute for Demographic Research, Konrad-Zuse-Str. 1, 18057 Rostock, Germany; 2 Population Research Centre, University of Groningen, PO Box 800, 9700 AV Groningen, The Netherlands

**Keywords:** Mortality Convergence, East German, Frail Individuals, Mortality Dynamics, Mortality Surface

## Abstract

**Background:**

Before German reunification, old-age mortality was considerably higher in East Germany than West Germany but converged quickly afterward. Previous studies attributed this rapid catch-up to improved living conditions. We add to this discussion by quantifying for the first time the impact of mortality selection.

**Methods:**

We use a gamma-Gompertz mortality model to estimate the contribution of selection to the East–West German mortality convergence before and after reunification.

**Results:**

We find that, compared to the West, frailer East Germans died earlier due to deteriorating mortality conditions leading to converging mortality rates for women and men already over age 70 before 1990. After 1990, the selection of frailer individuals played only a minor role in closing the East–west German mortality gap. However, our study suggests that, after reunification, old-age mortality improved quickly because the more robust population in the East benefitted greatly from ameliorating external factors such as health care and better living standards.

**Conclusion:**

Our results from a natural experiment show that selection of frail individuals plays an important role in population-level mortality dynamics. In the case of the German reunification, East German old-age mortality already converged before 1990 because of stronger selection pressure.

## Background.

A fundamental question in human aging research is what makes us survive to and at older ages. For more than 170 years, an ongoing increase in life expectancy has been observed [1]. Thus, survival seems to be shaped to a large extent by external conditions that improve the chances of reaching old and oldest ages [2–4]. Causal effects of changing extrinsic conditions on mortality are extensively studied in experimental situations among model organisms [5, 6]. However, these interventions are less applicable to humans and we therefore rely on natural experiments to derive causal relationships [7].

The separation and reunification of Germany provides us with a unique opportunity to assess external determinants of human survival to oldest ages on a population scale. Following experimental terminology, we have one population separated for four decades and experiencing different social, economic and political treatment. After reunification, East Germans received the same treatment as their West German compatriots. Mortality responded plastically to these changes in external conditions. During the separation, larger mortality differences emerged with East Germany falling increasingly behind West Germany [8]. After the fall of the Berlin Wall, this development was quickly reversed and life expectancy at birth reached the same level as in the West within two decades. However, mortality reductions occurred at a different pace among age groups. East Germans over age 60 contributed up to 80% to the life expectancy convergence and caught up earlier than younger ones [9]. Various studies sought to disentangle single factors that changed during the transformation and were particularly advantageous for older East Germans (for a review see Kibele 2012, Diehl 2004 or Luy 2004) [10–12]. They highlight that, apart from generally improved living conditions, women and men in the pension age benefitted from generous provisions of the West German social security system [13, 14]. In the first years after reunification, older East Germans enjoyed rising income levels and rapidly improving health care services. The currency union brought an overnight 5- to 10-fold increase in purchasing power, and the adoption of the West German retirement system resulted in a more than twofold rise in nominal pension income [15]. At the same time, investments in the East German health care system began to soar. Estimates reveal that the East German standard of health technology was lagging behind by 15 to 20 years at the time of reunification [16]. Medical infrastructure was outdated and hospitals in urgent need of modernization [17]. The improvements in quality and availability of medical care led to a quick mortality convergence particularly because of the reductions of circulatory diseases as the primary cause of death [18, 19]. Despite the importance of these changes that occurred after 1990, little research has focused on potential effects that pertain to the years before Germany’s reunification. Different studies show that mortality convergence or crossovers at higher ages among population subgroups are not necessarily a result of improving external conditions, but selection effects [20–22].

This research emphasizes that external determinants of mortality do not result in equal outcomes among heterogeneous individuals. The same treatment does not necessarily yield the same result even when we control for intervening effects. In mortality studies at the population level, this unobserved heterogeneity is explained by different susceptibilities to death among population groups [23]. Frail individuals are less resistant to mortality shocks, which decreases their survival probability over time. On the population level, this results in a relative increase of robust individuals with age. Only those with higher survival chances withstand mortality selection. Our study applies the selection into robustness framework for the first time to the German reunification. Herein, we seek to quantify to what extend selection effects during separation contributed to the old-age mortality convergence after 1990.

We assume that German mortality trajectories are shaped by selection effects. Eastern and Western German cohorts that were 60 years and older at the time of reunification faced a high mortality selection during their lifetime. They may have experienced the First World War, the Spanish flu, two economic crises during the 1920s, the Second World War and the subsequent rebuilding process. However, East German cohorts underwent additional social and economic hardship during the decades of German separation. While their western peers witnessed mortality declines caused by increasing wealth and medical progress, East German elderly experienced a marked disadvantage in terms of living standards, old-age care and health care delivery [24]. Based on this cumulative disadvantage, we assume that the number of frail individuals declined faster in the East than in the West where mortality conditions improved. The changing composition within these cohorts has an effect on the overall risk of death before 1990. We further hypothesize that the risk of death for Eastern cohorts with a comparatively higher number of robust individuals started to converge to the level of Western cohorts despite the comparatively worse external conditions. Hence, we expect that these cohorts benefitted from dramatically improving living conditions after reunification and reduced the comparative mortality disadvantage with their Western peers more quickly.

To account for selection effects based on deteriorating survival conditions, we estimate a gamma-Gompertz mortality model for East and West Germany. By referring to the mortality environment or general external conditions, we do not focus on one particular aspect. Instead, we refer to the general presence of resources that help to improve old-age survival like higher living standards or the availability of modern health care. We find that the changes in external conditions helped to lower the risk of death of elderly East Germans because of the selection against mortality that occurred already before reunification. Average frailty levels declined for cohorts who spent most of their life in the socialist Eastern part of Germany. This triggered a mortality convergence for the oldest ages before reunification and a quicker catch-up after the fall of the Berlin Wall.

## Methods

We follow a twofold analytical strategy to test our assumptions. By using a gamma-Gompertz mortality framework, we seek to estimate first whether older East Germans were more selected than West Germans, and second, whether these selection effects contribute to the observed mortality convergence. The gamma-Gompertz (ΓG) relative-risk model provides a sensible analytic framework to study humans as it captures the exponential increase in death rates between approximately ages 30 and 85, as well as the observed mortality deceleration thereafter [23, 25, 26]. The ΓG force of mortality $\overline{\mu}(x)$ is given by [27, 28].

1 $$ \overline{\mu}(x)={ae}^{bx}{\left[{\overline{S}}_c(x)\right]}^{\gamma }, $$

where *a* denotes the level of mortality at the starting age of analysis, *b* is the rate of individual aging (equal to the relative derivative of the Gompertz function $a{e}^{bx}$ *γ* is the squared coefficient of variation of the unobserved heterogeneity (frailty) distribution, and ${\overline{S}}_c\ (x)$ denotes the survivorship to age *x*. Each individual in the study population is assumed to have hidden (unobserved) susceptibility to death modeled by a random variable, named frailty [18]. Frailty accounts for the variability in individual lifespans due to unobserved, unmeasured or unavailable risk factors. Aggregate human mortality data, gathered by statistical offices and available for a list of countries in the Human Mortality Database, do not contain additional explanatory variables. As a result, when fitting a model to such data, including frailty is essential or else mortality estimates can be misleading [29]. For theoretical reasons [30–33] and computational convenience, frailty, defined as a random variable accounting for unknown individual susceptibility, is often modeled by a gamma distribution with an average of 1 at the starting age of analysis [23].

The ΓG model Eq. 1 captures mortality dynamics of cohorts. Each individual in a cohort follows a Gompertz mortality trajectory

$$ \mu \left(x|Z\right)=Z{ae}^{bx} $$

determined by one’s robustness to withstand physical decay (captured by *Z*). Every death of a frailer individual, i.e. an individual with larger value of $Z$, increases the proportion of robust individuals in a cohort and, thus, leads to a lower risk of death for the population. As cohorts age, the average and the variance of frailty at each subsequent age decreases. At a given age, comparing any of the latter frailty characteristics between two populations, can tell which population is more selected at this age.

Period mortality patterns result from the experience of the comprising cohorts at the age each cohort appears in the corresponding period. As the distribution of frailty varies by cohort and population, two populations can witness convergence or crossover of their mortality-rate patterns. This can happen if the lower-mortality population is represented at higher ages by a large proportion of frail individuals, while at the same age the higher-mortality population is represented by a small, but highly selected group of robust individuals.

Improvements since 1950 in age-specific mortality rates for a large group of industrialized countries [34] expose frail individuals to lower-than-predicted age-specific risks as they age. As a result, frail individuals survive to older ages. The ΓG model Eq. 1 is a uni-dimensional model for a cohort that fails to account for mortality changes that occur on a period basis and affect the survival of the members of the study cohort. This poses a problem if we want to measure the contribution of selection to the East–West German mortality convergence during the 1990s. As a result, we suggest fitting a gamma-Gompertz model to a mortality surface instead of single cohorts. The ΓG force of mortality on an age-period surface is given by [35]:

2 $$ \overline{\mu}\left(x,y\right)=\overset{\sim }{w}\left({x}_0,y\right){e}^{bx}{\left[{\overline{S}}_c\left(x,y\right)\right]}^{\gamma }, $$

where *x*_0_ denotes the starting age of analysis, $\overset{\sim }{w}\left({x}_0,y\right)$ measures the initial mortality level in year *y*, *b* accounts as previously for the rate of individual aging, ${\overline{S}}_c\ \left(x,y\right)$ measures the survivorship from *x*_0_ to *y* for individuals born in *y* − *x*, and *γ* equals frailty’s squared coefficient of variation. The latter is assumed to be one and the same across different cohorts, i.e. *γ*(*y* − *x*) ≡ *γ*. While this might be a strong assumption, perhaps not corresponding to empirical findings, it cannot be avoided: if in the model *γ* is allowed to vary by cohorts, Eq. 2 becomes statistically unidentifiable. Note that, formally, the term $\overset{\sim }{w}\left({x}_0,y\right)$ equals the product of *a*(*x*_0_, *y*), the initial level of the Gompertz baseline, and $\overline{z}\left({x}_0,y\right)$, the average frailty of all members of the (*y* − *x*)-cohort that survived to age *x*_0_. As a result, by estimating model Eq. 2 and $\overset{\sim }{w}\left({x}_0,y\right)$ (without loss of generality, as a smooth function) in particular, we are able to assess the joint (and statistically inseparable) effect of period mortality improvements and selection that has taken place within the (*y* − *x*) cohort until age *x*_0_ (see [35] for detailed discussion). Therefore, Eq. 2 does not permit estimating the “pure” selection effect on observed death-rate convergence.

To fit Eq. 2 we maximize a Poisson likelihood for the death counts *D*(*x*, *y*), assuming that $D\left(x,y\right)\sim Poisson\left(E\left(x,y\right)\times \overline{\mu}\left(x,y\right)\right)$ [36]. We take death counts *D*(*x*, *y*), exposures *E*(*x*, *y*), and the corresponding cohort survivals ${\overline{S}}_c\ \left(x,y\right)$ (for East and West Germany separately) from the Human Mortality Database (HMD) [34]. We consider a mortality surface for ages 75–99 and years 1980–1999 to capture the evolution (and the eventual convergence) of mortality in the decades prior to and after reunification. Note that the cohorts involved in this surface were born between 1891 and 1924.

To estimate the “pure” selection effect, i.e. the average frailty $\overline{z}(x)$ among survivors to age ${x}_0$ and above (for East and West Germany separately), we construct the ratio between $\overline{\mu}(x)$ and the Gompertz baseline *μ* ^0^(*x*) = *ae* ^*bx*^ as [23]:

3 $$ \overline{\mu}(x)=\overline{z}(x)\bullet {\mu}^0{(x)}^{23} $$

We fit a uni-dimensional ΓG model Eq. 1 for both sexes to the ages 75 to 99 for the year 1989 and 1995 and use the estimated *a*’s and *b*’s to reconstruct the corresponding ${\mu}_0(x)$. These ratios, yielding the average frailty $\overline{z}(x)$ at each age *x*, aid quantifying the contribution of selection to the East–West mortality differentials before and after reunification. The difference between observed age-specific relative risks and age-specific risks under the assumption of no selection, shows to what extent the mortality convergence is affected by different population compositions. There should be no difference between both ratios when the relative risk is unaffected by selection.

## Results

Our analysis yields two main results. We find that selection played an important role for the mortality dynamic among cohorts before reunification. Due to higher selection during the 1980s, the elderly population in East Germany was more robust than in the West. These different population compositions led to converging mortality levels before reunification. The mortality improvements after reunification are not predominantly driven by selection effects. However, the older East German population at the time of reunification consisted of more robust individuals that improved survival very rapidly.

**Fig. 1:**
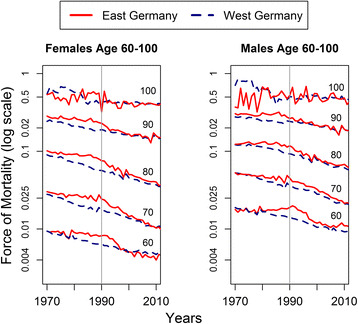
Period mortality for 10-year age groups in East and West Germany

**Fig. 2:**
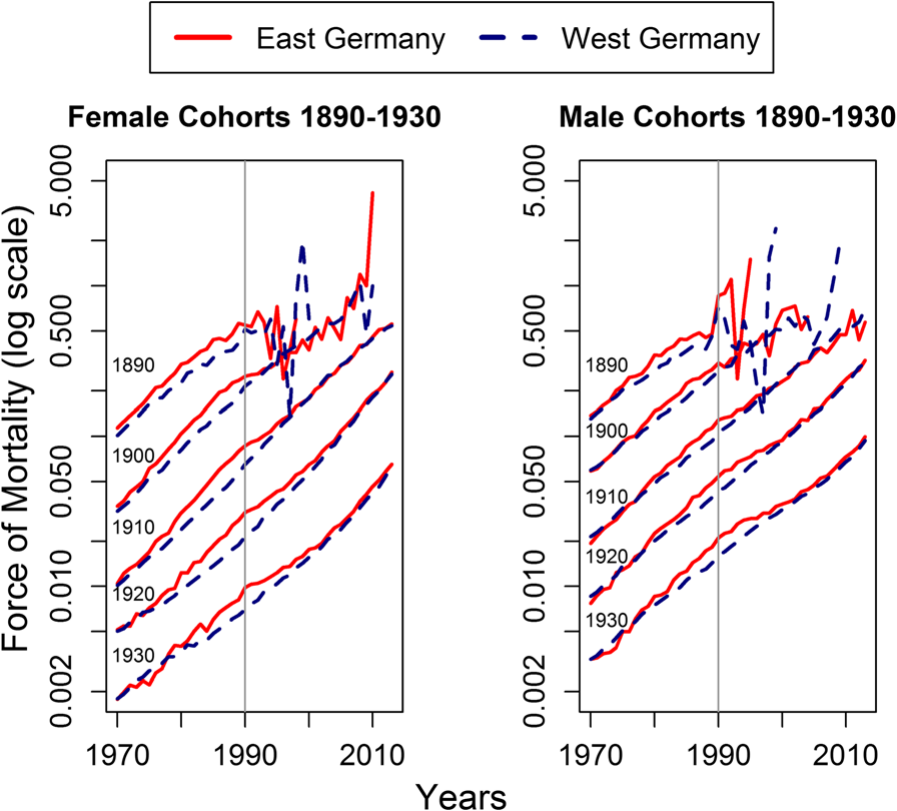
Cohort mortality for 10-year cohorts in East and West Germany

Figures 1 and 2 show the period and cohort mortality dynamics in East and West Germany from 1970 to 2010. It becomes apparent that since the 1970s, East Germans over age 60 lagged increasingly behind the West German mortality level. This holds true for both sexes and all age groups. Yet, the falling behind of the East is not due to a deterioration of mortality, but by the inability to keep up with West German improvements. Until the mid-1980s, mortality rates for East Germans remained rather stagnant but started to decline for males and females over age 70. The reunification of Germany brought mortality improvements for all age groups but an earlier convergence for older age groups than for younger ones. The exception from this pattern are East Germans over age 100 that were already as selected as West Germans before and after reunification.

The cohort perspective confirms the period results. We observe the same divergence and convergence patterns for the years before and after 1990. However, there is no sign for a catch-up of any cohort until reunification. This implies that the observed period convergence is not a result of improving mortality conditions in East Germany before reunification but rather a consequence of changing population compositions.

**Fig. 3:**
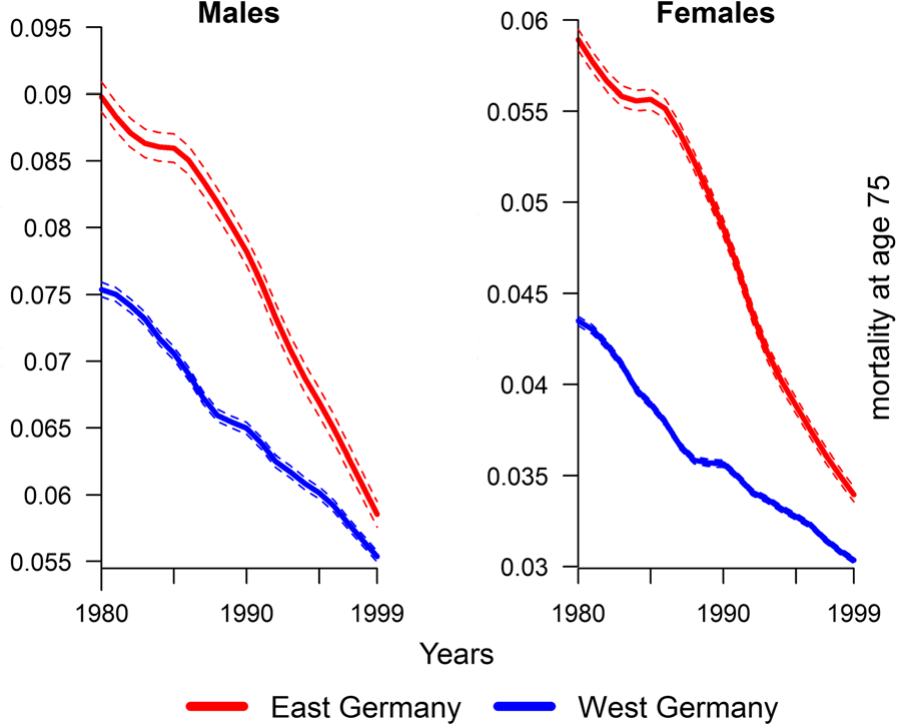
Evolution of $\overset{\sim }{w}\left(75,y\right)$ by gender in East and West Germany over the 1980–1999 period

Figure 3 synthesizes the period and cohort observations and shows the evolution of the baseline mortality, $\overset{\sim }{w}\left(75,y\right)$, in East and West Germany. Again, the baseline mortality for East Germans above the age of 75 started to converge to the West German level already in the mid-1980s. From a period perspective, this early catch-up is surprising as the mortality conditions for the elderly started to improve only after the German reunification in 1990. While the rapid decline of female and male baseline mortality during the 1990s may reflect these improvements, they cannot account for the changes in the 1980s. Thus, as we estimated $\overset{\sim }{w}\left(75,y\right)$ from a mortality surface, it suggests that the changes in observed mortality are based on different cohort compositions rather than on the improvement in period mortality.

**Fig. 4:**
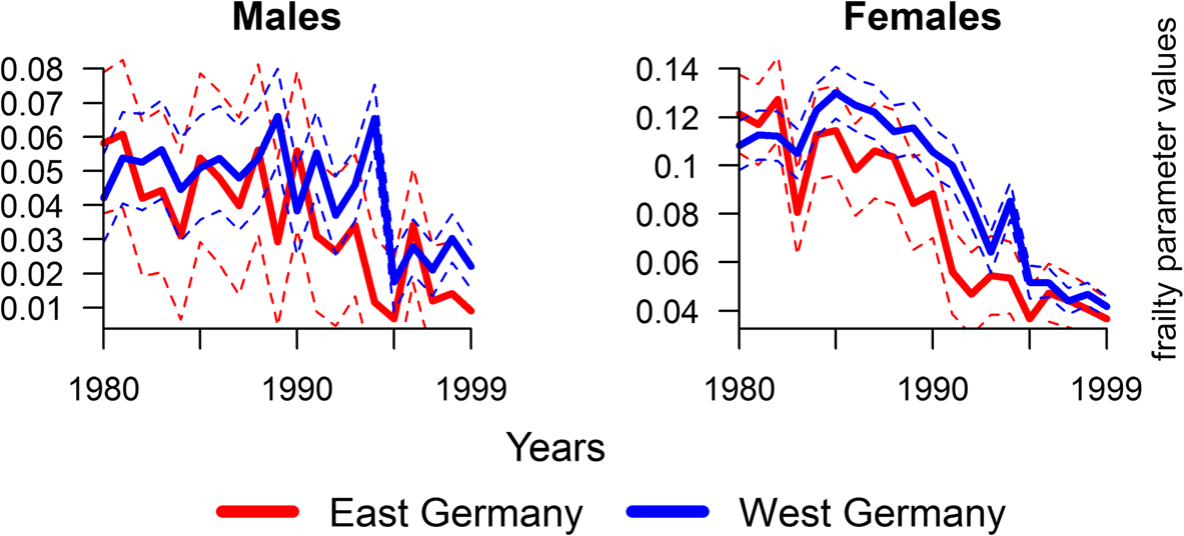
Estimated *γ*(*y*) with 90% confidence intervals by gender for East and West Germany in single years from 1980 to 1999

The squared coefficient of variation, *γ*, shows the degree to which cohorts in the East and the West were more selected during the 1980s and 1990s. Figure 4 shows that on average, cohort survivors in the East were homogeneously more robust than the same birth cohorts in the West. This is more pronounced among women than men and seems to be a result of stronger mortality selection of German men during the twentieth century. We observe a decline of *γ* for men only for the years after the German reunification. The squared coefficient of variation for women starts to fall already before reunification and converges to the West level at the end of the 1990s. Note that the 90% confidence intervals for *γ* in the East and West overlap for almost all populations (Fig. 4). On one hand, this suggests that the degree of heterogeneity in the respective East and West German cohorts is comparable. The wide confidence bounds, though, are an artifact of the small number of survivors to the oldest ages, especially for East German cohorts before the reunification. Note also that *γ*-values estimated for individual cohorts (rather than a mortality surface) are lower than the true *γ* as the uni-dimensional model is not able to capture age-specific mortality improvements as a cohort ages [29, 35].

Even though our estimations indicate that selection played an important role in the convergence of East German old-age mortality before reunification, it is less clear to what extent it explains the catch-up after reunification. The relative importance of selection becomes more apparent when we use hazard ratios for the observed and the baseline mortality for the years before and after reunification. The ratio between observed mortality in East and West Germany captures the relative risk to die for men and women in each age group in a given year. As this ratio includes the effect of different population compositions, we estimate a ratio that excludes the selection effects from the relative risk of death ratio. In other words, the baseline mortality ratio shows the relative risk between two populations under the rather unrealistic assumption of no heterogeneity within a population. Thus, the differences between the observed and baseline hazard ratios reflect the estimated robustness of the population and show the relevance of selection for converging mortality levels. This means that, for example, 85-year-old East German women in 1989 had a 1.3 times higher risk to die than West German women of the same age. However, their relative mortality risk would be higher (1.37) if their age group was equally selected as in the West. We compare the year 1989, the last year before reunification, with the year 1995, when Eastern female and male mortality for the oldest cohorts had already converged to the western level. Our results are not sensitive to the years chosen. Different combinations of years before and after reunification yield similar results.

**Fig. 5:**
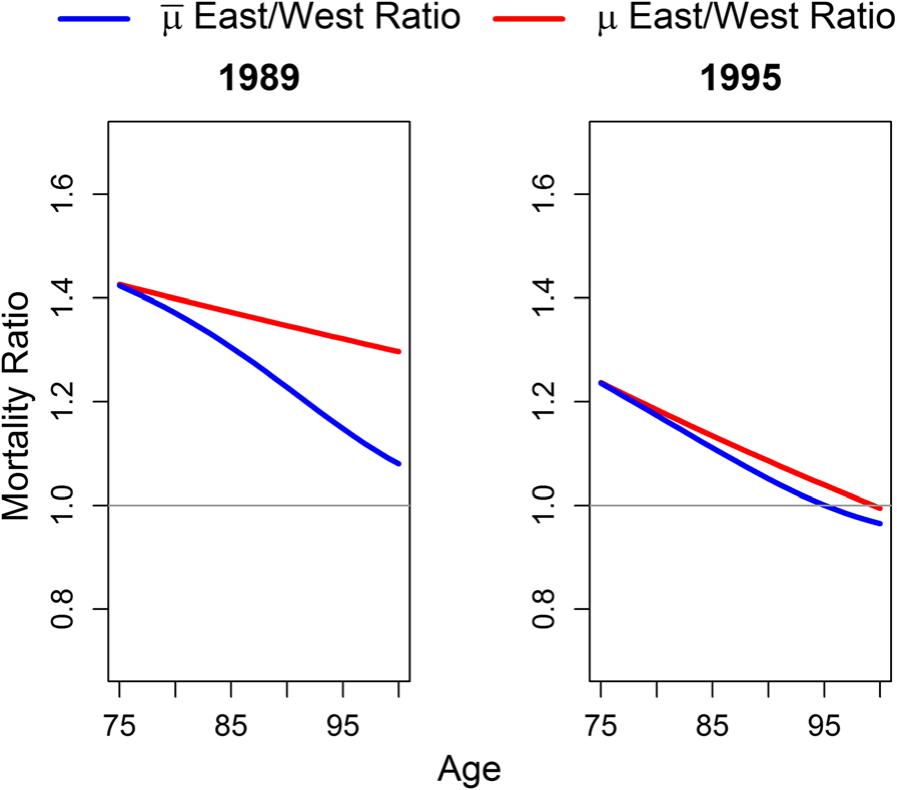
Female East/West ratios for baseline and observed mortality in 1989 and 1995

Figure 5 shows that East German women over age 75 before reunification had a 30–40% higher baseline mortality risk than women in the West, which may be caused by the worse East German mortality conditions. However, we find a decline in the observed mortality risk for the ages above 75 based on the more robust population composition in the East. This means that the population mortality risk declined by more than 20% for those robust individuals despite the worse conditions in the East. Without the increased selection pressure in the East, the relative mortality risks for older age groups would remain considerably higher and result in a persistent disadvantage over all ages rather than a mortality convergence. In 1995, the baseline mortality for all East German females has declined. Compared to 1989, women who are older than 75 years witness a general reduction of 20% in their baseline mortality risk. This risk even declines for older East German women, in particular, due to improvements in their living conditions. More robust individuals still converge earlier to the West German level. Yet, the selection contributes only 3% to the mortality convergence and is to a larger extent based on declining mortality disparities.

**Fig. 6:**
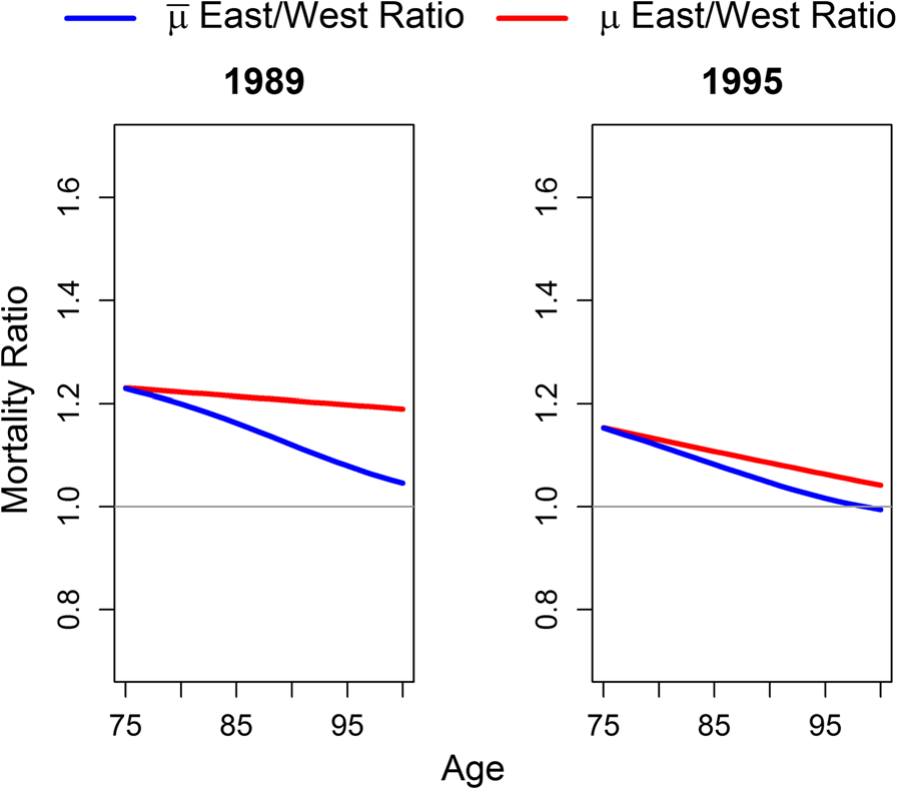
Male East/West ratios for baseline and observed mortality in 1989 and 1995

The male mortality ratios resemble the ones for females (see Fig. 6). In 1989, the baseline risk is “only” 20–25% higher for East German men. We find that selection played a substantial role in the observed pre-reunification catch up. The more robust population composition in the age groups above 75 reduces the observed relative risk by up to 20%. As for females, we find a gradual decline in the baseline risk for all ages above 75 in 1995. However, there is no general drop from 1989 to 1995. In 1995, men at the age of 75 could only reduce their mortality risk by 8% compared to the West German level. Still, we find that the post-reunification convergence is driven by general improvements in mortality conditions rather than selection of robust individuals. Selection among East German men reduces their mortality disadvantage by only 5% for the ages above 95.

## Discussion

Our analysis shows how selection effects can shape survival on a population level. We find that mortality among older East German cohorts converged to the Western level despite the worsening mortality environment before the fall of the Berlin Wall. At first, this is rather surprising. We would expect that diverging living conditions lead to increasing mortality differentials. However, survival on a population level is determined by an individual’s ability to withstand external mortality pressure. The observed mortality divergence during the 1970s and 80s reflects the growing differences in living conditions and the improved ability of older West Germans to withstand mortality pressure. Our study suggests for the first time that the mortality convergence of older cohorts before reunification and the quick catch-up afterwards were facilitated by a more robust population in the East.

Despite the importance of selection before reunification, its effect on the post-reunification catch-up is moderate. This holds particularly for men and is perhaps less surprising when we keep the equalizing living conditions and the mortality history of older East and West Germans in mind. Cohorts born before 1930 faced strong selection already before the German separation. They witnessed at least one of the two World Wars and the subsequent rebuilding efforts. Malnutrition, the Spanish Flu or the economic crises during the 1920s and 30s put additional selection pressure on these cohorts. Thus, we observe very similar frailty levels in both parts of Germany but higher selection in the East during separation.

Our analysis focuses on effects of generally changing mortality conditions on population compositions. Therefore, we cannot disentangle specific changes that led to improved survival after the German reunification. This potential shortcoming was addressed in various studies that identified single factors like health behaviors, income levels or health care availability that changed around 1990 [10–12, 15, 18]. Here, we sought to specify whether the observed mortality convergence is caused by these favorable changes or by selection against mortality before reunification. Another minor limitation arises from the data. East and West Germany had their last censuses in 1981 and 1987 respectively [37]. The next census was carried out in 2011 and has led to geographical and age-related correction of population counts during the last years [38]. An over- or underreporting of population counts for the years around the reunification could potentially distort the estimations of mortality rates. This source of bias for old age mortality is, nevertheless, minor for our analysis as noticeable disparities in reporting arose only for West Germany after 1997 [39]. A larger limitation lies in the cohort nature of the univariate gamma-Gompertz model that cannot account for period changes. As a result, the estimated degree of heterogeneity in every single cohort, captured by parameter *γ*, is lower or higher than the true one if age-specific mortality improves or deteriorates as the cohort ages, respectively.

We believe that our results aid understanding of how selection effects shape population-level mortality dynamics. Our study suggests that survival to and at oldest ages is to a large extent shaped by extrinsic mortality conditions. It seems that human survival to and even at oldest ages can be remarkably improved by improvements in the mortality environment despite unfavorable conditions earlier in the life course [40].

## Conclusion

Mortality convergence on the population level is often caused by selection against frail individuals. Our study adds to this literature and estimates for the first time to what extent selection effects contributed to the converging mortality trajectories in Germany after the fall of the Berlin Wall in 1990. Previous studies attributed mortality declines among older East Germans to period effects like improving health care or a general rise in living standards. We use a gamma-Gompertz model to show that mortality of older East Germans started to converge to the western level already before reunification despite overall worse living conditions. Without selection, the relative risk of death in the East would have been up to 20% higher than it was actually observed. The rapid mortality catch-up after reunification is predominantly driven by changes in East German’s living conditions and to a lower extent by continued selection pressure. Thus, our study confirms the conclusions from previous studies that use the German reunification as a natural experiment and highlight the importance of period effects. However, it also suggests that the remarkable improvements in old age mortality were potentially facilitated by a more robust population composition in East Germany.
